# A Novel Privacy Preserving Scheme for Smart Grid-Based Home Area Networks

**DOI:** 10.3390/s22062269

**Published:** 2022-03-15

**Authors:** Wajahat Ali, Ikram Ud Din, Ahmad Almogren, Byung-Seo Kim

**Affiliations:** 1 Department of Information Technology, The University of Haripur, Haripur 22620, Pakistan; wajahat.haripur@gmail.com; 2 Department of Computer Science, College of Computer and Information Sciences, King Saud University, Riyadh 11633, Saudi Arabia; ahalmogren@ksu.edu.sa; 3 Department of Software and Communications Engineering, Hongik University, Sejong 30016, Korea

**Keywords:** aggregation, authentication, key management, privacy, smart home, smart meter

## Abstract

Despite the benefits of smart grids, concerns about security and privacy arise when a large number of heterogeneous devices communicate via a public network. A novel privacy-preserving method for smart grid-based home area networks (HAN) is proposed in this research. To aggregate data from diverse household appliances, the proposed approach uses homomorphic Paillier encryption, Chinese remainder theorem, and one-way hash function. The privacy in Internet of things (IoT)-enabled smart homes is one of the major concerns of the research community. In the proposed scheme, the sink node not only aggregates the data but also enables the early detection of false data injection and replay attacks. According to the security analysis, the proposed approach offers adequate security. The smart grid distributes power and facilitates a two-way communications channel that leads to transparency and developing trust.

## 1. Introduction

The term power grid is commonly referred to as an electricity distribution system that supplies energy to a territory. A power grid actually comprises power generation, distribution, and transmission [1]. The traditional power grid just supplies energy to the consumer which results in simplified management but at the cost of short falls when supply and demand do not catch up. There are certain limitations of the traditional grid such as the losses at transmission lines and lack of information, or we can say that lack of demand knowledge, which further leads to inefficient power management [2,3]. For example, the traditional grid feeds constant power during peak and off peak hours. To overcome the limitations of the traditional power grid, certain changes must be made in traditional grid [4]. The power sector needs to be revolutionized to meet the needs of modern living. A smart grid promises to replace the traditional grid with better performance and is also open enough to meet the upcoming revolution in the power sector. The SG consists of a power generation unit, power transmission and distribution units, smart meter (SM), smart homes, smart energy management systems, and smart appliances [5]. The communication among power generation, transmission, distribution, and customers is usually a two way communication managed by the CC and service provider enabling real time communications between consumers and the utility/service provider [6,7].

An SM senses the energy consumption of a home and sends it to a substation or the gateway or control room of that region. There may be a number of devices between the SM and service provider. The SM reports the energy consumption about every 15 min to the service provider [8]. The control room receives data from all the SMs in the neighborhood and transmits the combined energy usage report to the control center. The control center uses the consumption report to run load management and power distribution and uses the information for billing purposes [9]. Abbreviations contains all the terminologies and their definition used in the paper.

### 1.1. Architecture of Smart Grid

Smart grid refers to the electricity distribution network that uses communication channels to detect any change in local power usage and acts accordingly without external interference. It uses smart home appliances, SM, and green energy resources. The smart grid utilizes a two-way communication channel and allows consumer to interact with the grid. It facilitates the consumers, service provider, and government establishment by overcoming the drawbacks of traditional grid. It reduces the energy consumption and decreases the consumer’s cost of electricity by smart means.

### 1.2. Working Architecture

The smart meter works inside a HAN as follows:

- The SMs are installed at the home, offices, and factory premises. The SM communicates to the local control center (CC), which is the nearest data gathering center. An SM can provide instantaneous consumption, cumulative energy, time of day energy data, and maximum demand (in kW).
- The local data center transmits information gathered from SMs in a locality to the data center at the utility provider or any third-party service provider using wired or wireless means.
- The data at the utility provider side can be accessed using a web portal. The utility providers gather data from local CC in real time and process it. It reports any tampering of meters, billing information, energy usage, load status, etc.

### 1.3. Our Contributions

The communication that makes up the grid smart is one of the obstacles that the smart grid deployment faces. To date, several schemes have been proposed and review surveys have been published, for example, [10,11,12,13,14]. Most of these schemes highlight either communication, technology standards, and infrastructure or home energy management and security. To the best of our knowledge, however, privacy is a big issue that still has to be thoroughly examined.

Since the smart grid is designed to facilitate its consumers, keeping the privacy of end users in a HAN is important. To date, a few schemes have been proposed, such as [15,16,17,18,19,20,21,22,23] to create a safe communication route over vulnerable public networks. These schemes are aimed at establishing a framework that can potentially protect end users’ privacy. The majority of the plans cover the smart grid in basic terms, but they leave out smart homes and HANs. As it runs in the field, a HAN is the most vulnerable to cyber threats, theft, and data tampering. Because a consumer may be unaware of cyber security standards, it is critical to have built-in security to protect HANs from various cyber threats. In short, the issues associated with smart homes are rarely explored in published articles. This paper discusses smart homes and gathers the prominent articles in this domain, and proposes a novel privacy preserving scheme. Here, we highlight the following points:

- The development of smart grids is discussed along with the architecture of smart grids.
- Data aggregation, privacy preservation, key management, and user authentication are discussed within the scope of a smart home.
- A comprehensive literature review along with the pros and cons of existing schemes is discussed in addition to presenting some advanced literature to solve these issues.
- The paper tries to focus on smart homes and the privacy concerns of consumers along with discussing the future directions for faster transformation from traditional to smart grids.
- Finally, a privacy preserving data aggregation scheme is proposed for HANs that gathers the readings from all home appliances at the sink node, performs an early stage fault tolerance and aggregates the received reading data into one, and sends the result to the SM for further analysis.

The remainder of the paper is laid out as follows: The smart home is introduced in Section 2. Section 3 discusses the notion of privacy, its parameters, goals and attacks, and the threats to privacy. Section 4 presents a comparative analysis of advanced privacy-preserving techniques, including their benefits and drawbacks, as well as countermeasures. Section 5 describes a privacy-preserving data aggregation technique for fault-tolerant smart homes. Section 6 examines the proposed scheme’s security measures, followed by a performance evaluation in Section 7. Future study directions are discussed in Section 8, and the work is concluded in Section 9.

## 2. Smart Home

**Figure 1:**
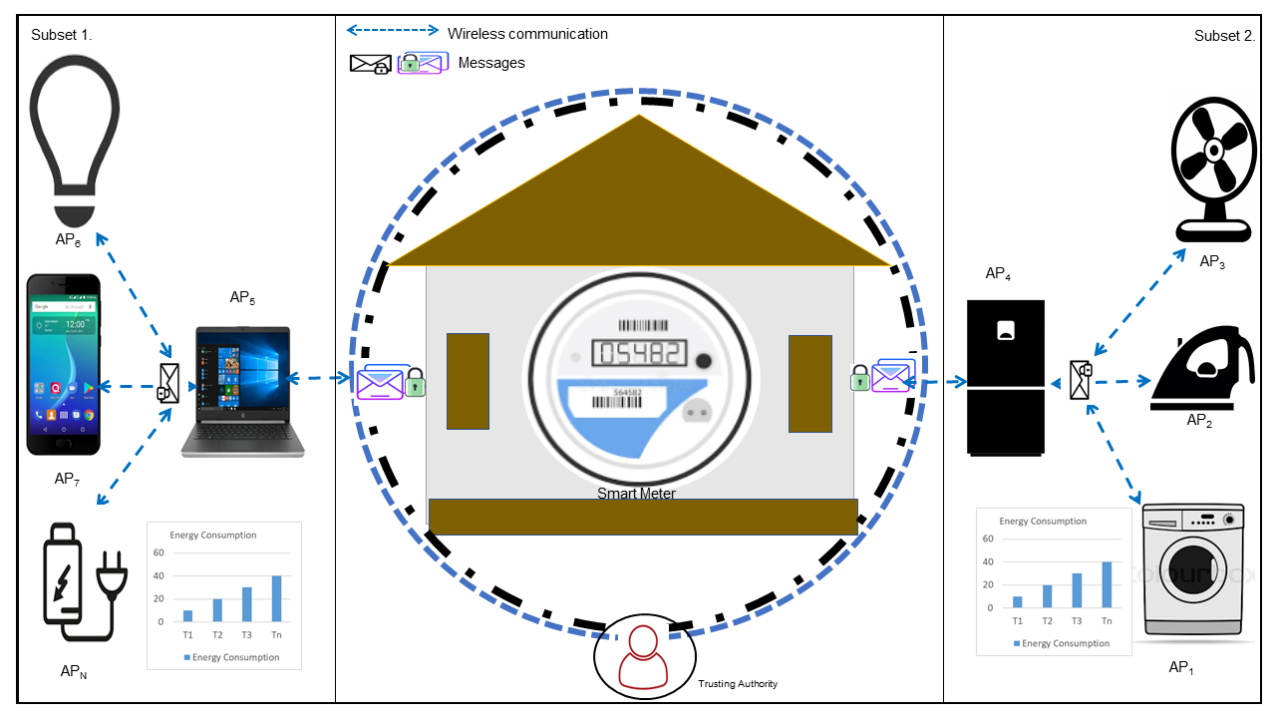
A home area network.

A smart home consists of an SM and various appliances. Appliances may be a low voltage devices or high voltage devices which aggregate their energy consumption and send information to SMs, as shown in Figure 1. The SM receives energy consumption from appliances and forward to utilities for further processing [24,25]. Home energy management system (HEMS) is an automated system consist of hardware and software which controls and monitors the various devices and their operations. Users manually manage and control the electricity generation and production [26]. Different hourly block rates are offered for 24 h. Needless devices are automatically turn off with a short notification. Demand side management, demand response, direct load control, real time pricing, time of use, and real time peak pricing are recent examples of HEMS [27].

### 2.1. Smart Meter

An SM is an electric meter that performs the following functions: (i) measuring energy consumption, (ii) measuring energy consumption, (iii) report consumption data to meter management system, (iv) receiving electricity consumption cost or control signals, and (v) inform all the home appliances [28]. The SM data is one of the major sources of information similar to other actors, e.g., distribution system, transmission system, and generation sources for smooth running and construction of smart grid. As per European commission report around 80 percent of energy meters will be replaced with SM in 2020 [29].

### 2.2. Home Appliances

Home appliances are household devices installed in user home or apartments. These devices are connected to SM for monitoring and reporting [30]. Users can schedule appliances per use. In [16], the appliances are classified into four groups: Group 1 includes light load normal appliances, e.g., light bulbs and phone chargers; Group 2 consists of nonstoppable home appliances, e.g., microwave ovens, Group 3 comprises schedule-oriented appliances, e.g., washing machines and heaters; Group 4 includes electrical vehicles. Consumer can also schedule their appliances with different hourly changing rates to control their electricity cost [16]. Appliances monitor their energy readings and send to the SM after every time interval, which is usually 15 min duration [28].

### 2.3. Wireless Sensor

With the invention of new technologies, wireless sensors are being used in industries, health care, education, and utility grids. Due to their sensing capabilities, it makes them able to interact with machines, devices, and various appliances for controlling and monitoring [17,31]. Similarly, with the deployment of smart grids, wireless sensors are being deployed in smart homes and also at utility. Wireless sensor performs conversion of analog signals to digital, analog signal processing, transformation of information via bidirectional bus, manipulation of sensor derived signals, and addressing [32]. In a HAN, kitchen appliances, heating system, security frameworks, lighting system, theater setups, and water and sewage systems are totally instrumented with wireless sensors performing various operations. Access to these systems is through a home management system (HMS), which could be through the Internet or a cell phone application [32,33].

### 2.4. Consumer

Consumer is the main stakeholder for which the smart grid is designed. Consumers can schedule energy consumption, generate green energy for themselves, and store energy for future purposes [34]. Consumers can control their appliances’ function and information flows, such as home automation, home energy management system, and industrial automation system [24].

### 2.5. Advanced Smart Home Applications

To upgrade the functionality of conventional grids, smart grids have introduced various new applications in smart homes, e.g., energy generation and storage and demand response. Smart grids have enabled the consumer to control their electricity bill. Other benefits that smart home applications have provided are scheduling power usage during the on/off peak hours, they can demand extra energy from the grid in advance, and, if required, they can also feed any green energy generated back to the national grid.

#### 2.5.1. Two-Way Communications

A smart grid establishes a communications channel between consumers and service providers. A consumer can request peak and off-peak hours tariff rates to schedule the electricity usage appropriately. The SP can also obtain a future consumption forecast and can therefore control the energy production [35].

#### 2.5.2. Renewable Energy Resources

With renewable energy resources an individual home can generate its own energy using mostly solar panels, but also with windmills or biogas. A consumer uses part of the energy for their own purpose and can feed the extra energy into the national grid. Hence, a consumer can also participate in the national grid and play a useful role for the national cause [36].

#### 2.5.3. Energy Generation and Storage

With the invention of energy renewable resources such as solar, biogas, wind, and electricity storage sources such as electrical vehicles, smart transformers, and appliances, many home users generate electricity using photovoltaic panels for their daily use and sell extra electricity to the national power grid [35]. In each area, every application of SG is based on necessities such as voltage support, power quality, and service reliability [36]. However, smart grids have a serious issue with generating and storing electricity and, similarly, with the evaluation of the distribution system and integration of the evaluated grid components. Currently, gas and diesel generators, tides, and solar and wind are conventional energy generation resources, which provide power whenever natural resources are not available. The authors of [37] proposed optimization techniques for domestic users to control the operations in a HAN. Similarly, in [38], the integer-programming model is presented for electricity storage based on electric vehicles and photovoltaic panels.

#### 2.5.4. Demand Response

With the rapid increase in population and wireless devices in HAN, demand for electricity has also increased. In a conventional grid system, it is hard to accommodate the electricity needs because conventional systems have various challenges, such as maintenance, erection, operation, and design [39]. This strategy is used to control or reduce the electricity consumption at peak hours. It does not only reduce the use of electricity consumption at peak hours but also reduces the electricity consumption cost. Different rates have already been offered for various hours [18,19].

## 3. Privacy

Smart grids are a promising technology describing electrical power infrastructure for transmission and distribution with integrated information and communications technologies [40]. The purpose of a smart grid is to bill the customers accurately and manage and distribute electrical energy in an efficient way. In a smart grid, an SM is the key entity. When an SM is deployed, the concern regarding meter tempering and consumer privacy is raised. There is a need for legislation of SMs. In compliance with privacy requirements, certain properties ensuring the privacy are confidentiality, integrity, authenticity, and availability [24,41]. An SM is prone to data tempering where an adversary can invade the SM. If an SM is compromised, it is then easy to access a cryptographic key. By exploiting a common vulnerability, a large number of SMs can be compromised and can result in manipulating real-time consumption. Therefore, a scalable access control is needed to prevent meter compromises and make sure that any stored information is used for the purpose of billing operations and other value-added services [42]. The major benefit of an SM is accurate billing, but the frequent sharing of consumption information with the utility might leak some private information. In order to protect billing information techniques—e.g., battery management—a zero knowledge homomorphic encryption technique is proposed in [43].

### 3.1. Privacy Goals

An SM collects the energy consumption from home appliances usually after a 15 min duration. An SM then generates a consumption report and sends it to the utility company. There might be an adversary peeking at energy consumption of a smart home that can further predict the lifestyle and routine of a homeowner. This poses a threat to security as well as privacy of the smart home. Therefore, to preserve privacy and security, energy consumption is encrypted either at the appliance level or before it leaves the HAN. The energy consumption sent by the HAN is further processed and the CC receives energy consumption details from various SMs. Each meter reports either its own consumption or an aggregation technique is used to sum-up the consumption reports from all meters in a sub-region/zone and send a bulk consumption report after the aggregation. Based on this, the CC generates monthly bills and maintains a profile picture of that region to show that how much electricity consumption is required [44]. The CC continuously collects consumption details from different devices. The received consumption is encrypted or aggregated because if an intruder changes the message, it could easily be identified [20]. This section describes various privacy goals.

#### 3.1.1. Confidentiality

Home appliances send their energy detail to aggregator, SM or SP. This detail may reflect a users’ personal profile. Privacy and confidentiality are interdependent of each other [45]. By securing messages from unauthorized access, privacy of home incumbents will be retained. If privacy of home users is compromised, then confidentiality is automatically violated. To ensure confidentiality, various schemes for HAN are employed, e.g., homomorphic encryption, blind signature, in-network aggregation, etc. [16,21].

#### 3.1.2. Integrity

Smart appliances continuously send consumption patterns to an aggregator or SM after an interval. It may be possible that an aggregator or an SM are physically secure but vulnerable to different attacks such as man-in-middle attacks, alteration attacks, or replay attacks [46]. If integrity is compromised, precious information will be compromised and wrong decisions for managing and controlling the network might be made. Integrity refers to the message sent by sender; the same message without any modification is received by receiver. The following schemes are used for HAN data integrity: message digest, MAC, digital signature, and H-MAC [16,22].

#### 3.1.3. Anonymity

Anonymity refers to the situation where the real identity of a person is kept secret. During sharing of secret control signals or reading, a device may protect their real identity from other appliances or devices [47]. Even an appliance or SM cannot recognize other devices communicating with them in a HAN. The purpose of anonymity is to hide one’s identity from appliance to appliance, appliance to SM, SM to SP, and SP to appliance. Various techniques for anonymity include PALK, ASF, and TAI [48,49].

#### 3.1.4. Availability

Availability indicates that data, applications, and systems are available to end-users when they are required. Availability is compromised if someone pretends to be an authorized user to access the system and make the network busy [50]. Distributed denial of service (DDoS) is the most basic availability attack. In DDoS attacks, the incoming traffic is originated from multiple sources; therefore, making it difficult for offensive measures to identify a single malfunctioning device. A DDoS attack in IoT devices happens due to lack of security measures. The alternating direction method of multipliers [51] and honeypot game models [52] are used to protect the systems from DDoS attacks.

### 3.2. Cyber Attacks on Smart Homes

In a HAN, various heterogeneous devices are connected to each other. The devices are interoperable and are managed remotely. An adversary is always searching to find an entry point to enter the network for different attacks.

#### 3.2.1. Impersonation Attack

Each device’s status—ON/OFF—is saved in the SM memory. Every 15 min the appliance sends its consumption to the SM. If an appliance is compromised and impersonates another device, this can result in a false reading for a time period unless it is detected and recovered, for example, if AC is switched ON and impersonates a fan or light bulb and vice versa, then it has a huge impact on power billing [53]. Even if an appliance impersonates itself as the SM and requests that the other appliances send consumption reports every 15 min, the result could be dangerous and can lead to some disaster or might lead to electricity theft [54].

#### 3.2.2. Eavesdropping

Smart grids are meant not for the electricity supply from grid to home or home to grid but also as a communication channel between a smart home to the SG and also sends various control messages and forecasts the power demand in advance [53]. If an adversary eavesdrops or sneaks into someone’s SM, he or she can easily know the homeowner’s routine/lifestyle, living habits, and interests (tuned TV channel) as well as the time they go to work and when a person is at home or not. This information may result in compromising the customer’s privacy and also can also be used to plan for theft and other activities.

#### 3.2.3. Replay Attack

Smart homes and SG are continuously communicating and sharing information about electricity usage and forecasting the future power demands. If there is a compromised appliance or SM, an adversary can see the consumption report and can replay an old report in place of the current report and can also change the demand-to-supply report or even replay an old control message. For example, if extra power is demanded or an appliance asks to be scheduled for off peak hours, a replay attack could alter the demand to low power or the appliance can be switched ON at once and cause an inconvenience [55].

#### 3.2.4. Alteration Attack

An alteration attack happens when the HAN, an appliance, or the SM is compromised and an adversary maliciously alters the consumption report or forges a message. The forged message or consumption report can lead to false execution, for example, if a message is sent to set the oven to 120 °C but when altered sets the water heating system to 120 °C then it might lead to injuring a person at home or can also lead to system failure or short circuit. Even if a consumption report is forged, it may cause the customer to pay for electricity that he has not consumed [56].

#### 3.2.5. Message Modification Attack

Communication is a key way in which the SG differs from a traditional grid. If there is an adversary between SG and HAN, it can modify the messages sent to or received from the SG/HAN, which may result in a trust deficit between the working entities and thus leading to serious damage at either side [23].

#### 3.2.6. Energy Import/Export Attack

The SG allows for distributed power generation, where a consumer can install the renewable power generation resources at the consumer’s premises. It feeds the surpass energy into the national grid and can also demand extra energy resources from the grid when needed [23]. For example, an adversary demands the energy import from the grid, which is not needed, and exports the energy from home to the grid even when it is needed at home [56]. Similarly, if a plug-in electrical vehicle is charged and imports unnecessary energy from the grid, which is not needed at peak hours, it can lead to power shortfalls and load shedding.

## 4. Advanced Privacy Preserving Scheme and Its Countermeasures

In this section, we study the latest privacy preserving schemes related to user authentication, data aggregation, key management, and CIA triad.

In [32], the authors have reviewed security issues related to smart homes. The purpose is to portray the scenarios that pose a threat to smart homes, which are an essential part of the smart grid. The smart grid security objectives adopted in this paper are confidentiality, integrity, availability, authenticity, authorization, and nonrepudiation attacks. Furthermore, in [32], the authors examine potential cyber and physical security threats in terms of security objectives.

The communication infrastructure of the smart grid, while considering reliability and challenges to security in the smart grid, is provided in [57]. In [58], an emerging technology, i.e., software defined network (SDN), is discussed. A complete overview of the HEMS literature with reference to main principles, setups, and enabling technologies is offered in [59]. The scheme in [60] comprises existing architectures, applications, and prototypes of IoT-assisted SG systems, and provides an overview of IoT-assisted SG systems.

In the studied literature, the following points have been observed:

- The security challenges of the smart grid are discussed and threats are evaluated.
- The existing architectures, prototypes, and communications challenges are discussed.
- The challenges related to interoperability of various technologies at the hardware level of the smart grid are discussed.

In [61], big data collection and management is surveyed. The authors have used an analytical method to study big data and its applications associated with smart grids. The paper gives an insight about the sources of big data in smart grids and real-time processing to predict a pattern for decision making. In [62], a detailed survey on the future wireless communications systems is performed. The authors have reviewed the energy utilization, redistribution, and trading. The authors have performed a comprehensive study of the current literature and have observed the concern about security vulnerabilities of smart grids. In [63], with the use of new IoT technologies, an overview of smart grid security improvements and weaknesses is provided.

### 4.1. Data Aggregation

Data aggregation is the process of gathering data from several sources and combining it into a variable or report. In smart homes, various appliances are connected to an SM and send their demand/consumption report to the SM. It creates a communications overhead and privacy hole [64]. To avoid this issue, an aggregator is used that collects messages from various appliances and aggregates them into a single message. The following techniques are discussed for data aggregation while preserving the privacy.

In [22], a scheme has been presented, which is based on incremental hash operation. This scheme reports the cost to the operation center instead of energy consumption readings. After an interval of time, the SM calculates the cost of the recorded reading using hash function and sends it to an operation center. The operation center first receives all the consumption costs from different residential areas and then aggregates them for forwarding to utility providers for the verification of integrity. Utility providers sum up all the received values and compare it with the power distribution for that time interval to validate the integrity. If the value of cost and distribution is not equal, the entire consumption reading is discarded automatically.

A framework based on Shamir’s secret sharing is proposed in [65] in order to effectively reduce computational overhead and dependency on a single dedicated aggregator. The scheme also prevents the electrical utility from linking its data to a single SM. The architecture describes that the area under the supply of one service provider is divided into subregions. Each SM divides its reading into shares and connects it to several aggregators. The scheme masks the SM form the utility by sending the aggregated reading and reduces the dependency on a single aggregator.

An in-network data aggregation scheme is proposed in [30], which aggregates the data hop by hop. Each appliance has its own chip code and spreads the energy consumption using these chip codes, which are sent to the SM after every time interval. These chip codes are unique among appliances. The SM can extract each appliance’s consumption by knowing the chip codes. Since each appliance has its own chip code, any malfunctioning appliance cannot alter the consumption of other appliances.

In [66], a multidimensional aggregation scheme is used to save the communication bandwidth and increase the computational speed of the SM. There is a gateway between the CC and HAN, which receives the encrypted data from a large number of SMs and then aggregates the data before sending it to CC. A TTP is used to mask the gateway from HANs to avoid any mishandling. Any failure or attack on the TTP end can lead to a serious disturbance in communications between the CC and HAN.

**Summary:**Table 1 provides a detailed aggregation summary of the above analyzed techniques. It is perceived that the majority of the aggregation steps are performed by a separate third party device or CC [22,65,66,67]. Similarly, in [65,66], the selection of devices for aggregation and their group header nomination also increases computation overhead. The authors of [67] assume that all entities taking part in the communications are secure and resistant to tampering and modification attacks.

### 4.2. CIA Triad and Anonymity

In this section, we present schemes that ensure CIA triad and anonymity while preserving the privacy of HAN. A scheme proposed in [68] divides the users of a residential area in subsets based on the energy consumption ranges over a period of time. The energy consumption is then summed up for each subset. The TTP and Paillier homomorphic schemes are used to ensure the privacy of data. However, a damaged SM may not report the data correctly and the malfunctioning or misuse of TTP can lead to serious concerns regarding the authenticity of aggregation reports.

In [69], a Q-learning technique, which is based on artificial intelligence, is proposed and presented. The structure is that there are three kinds of information shared between a HAN/BAN or SCC: control flow, data flow, and power flow. Smart appliances and SMs constitute the HAN. Different HANs that are in the same building constitute a BAN. The regional power supplier which manages multiple BANs is called NAN. The NAN sends information such as dispatch instruction, billing, real-time reporting, and uploads the data to the SCC. Before sending data to the control center, the data is distributed to uniformly random secret shares. SCC outsources information to professional cloud server operators to train the Q-Learning model using edge computing. The secret shares are randomly distributed so that cloud servers could not obtain the information. However, if the two servers collude, then it can be a very serious privacy breach. The scheme also has it own protocols for selection and addition and subtraction but, as we know, the honest but curious entities in the network can access the information from the secret shares anytime.

Similarly, in [16], a homomorphic scheme is proposed for smart homes, which consists of home appliances, SM, and a third-party aggregator. The third party aggregator assigns an ID to every appliance at the time of installation. All appliances in a home are similarly arranged in a sequence order as per given IDs. All appliances report their consumption report to an SM. Before sending their consumption, they add homomorphic features and forward data to the aggregator for the current round. The aggregator appliances sum up all received readings, encrypt it with SM’s public key, and send to the SM. The SM authenticates the aggregator appliance using a private key.

The SM encrypts the consumption and gives the identity to the SS. After verifying the identity, SS generates the group blind signature and generates the tags for each data block which the CC acquires and matches with the corresponding data block. In this way CC verifies the data integrity. The author supports the scheme by following that if an adversary or SS somehow can obtain the encrypted consumption but could not obtain the CC’s private key. This is because in order to guess the private key prime numbers must be used and exact prime numbers are difficult to match in a polynomial equation. Thus, the possibility of compromising the CC’s private key is almost negligible. However, the CC is assumed to be honest in this scheme [21].

In many privacy preserving schemes, TTP is certification authority to generate public and private keys. To avoid TTP, Xiaoli et al. presented a secure privacy preserving scheme. At the time of physical configuration each SM is assigned an ID by the CC [70]. The same ID is also registered with the CC. Every time the CC sends a request message to the SM for sending the energy consumption pattern, the request message includes SM, CC ID, and the key material. Using ID and key material, the SM first generates a random number and then a secret key. The SM will encrypt the energy consumption report by using their secret key and current time stamp. The encrypted message is then forward to CC for identity verification and decryption. The CC first verifies the SM identity by its ID and then decrypts the message using the same secret key.

**Summary:** PPMA [68], LiPSG [69], and lattice-based homomorphic schemes [16] provide confidentiality and integrity, but not anonymity and availability (see Table 2). PPMA and lattice-based homomorphic schemes are resistant against passive and active attacks, but blind signature [21] fails to do this. Similarly, [16,21,68,70] do not update their encryption key.

### 4.3. User Authentication

Authentication is a process of associating the incoming activation requests with the already set authentication rights [71]. These authentication rights are stored in file systems or databases. When any device sends its consumption to the SM based on the designed schemes, the system allows or denies the request.

In [72], a scheme is designed, which is based on elliptic curve cryptography and consists of three phases, i.e., system-setup phase, registration phase, and key agreement and authentication phase. Initially, in the system setup phase, the trust anchor shares the system parameters using an elliptic curve and publishes these parameters. In the registration phase, the trust anchor generates the private key for both the SM and the SP using Schnorr’s signature. After registration, the SM and SP communicate directly without the involvement of a trust anchor. In the last phase, the SM and SP automatically generate a session key and authenticate each other via session and private keys.

In [15], data source authentication and data aggregation are performed for a particular residential area over a defined time period while ensuring the privacy of each user’s data aggregation and fault tolerance. This scheme provides a high level of control over data collection and the processing phase in addition to verifying the integrity of the data and validates the data source.

To eradicate computations and communication resources, a lightweight authentication scheme is presented in [73], which is based on a physically-unclonable function. Before any communications, the SM and neighborhood gateway authenticate each other. The SM sends the ID to the neighborhood gateway. The neighborhood gateway checks the SM ID in its database and creates two random numbers, concatenates these numbers with the time stamp, and the result is XoRed with R-response and sent to the SM. The SM authenticates the neighborhood gateway for further communications.

In [21], an SG is divided into three layers. The CC lies in the middle layer and is responsible for generating system parameters, user registrations, and the verification of data. The SM is placed at the lowest layer and monitors/sends real-time consumption; therefore, it is prone to data being tampered or manipulated.

Similarly, in [74], elliptic curve cryptography is used to authenticate the entities in the SG to preserve the communication between them over a public and insecure channel. First of all, TTP generates all system parameters and then authenticates the SG device and UC in an offline mode. The scheme is robust against certain attacks; however, the pre-loaded system information may affect the computation power of the smart devices.

In [75], the authors have proposed a scheme to achieve anonymity for the SM to avail all the services provided by the UC, without the involvement of TTP. TTP is only responsible for the registration phase, and its role is limited. The SM is supposed to send the consumption report and control signals to UC, which is an aggregator as well as controller for monitoring the energy consumption trends. Authentication will take place between the UC and the SM.

**Summary:**Table 3 contains a summary of the analyzed techniques for authentication. In [21,74,75], the SM and CC authenticate each other but the appliances are not authenticated. In [72], only the CC performs authentication; the SM and appliance are just relay nodes. Similarly, [74,75] are prone to cyber security attacks and require higher computational cost.

### 4.4. Key Management

A key is a bit of code encrypting and decrypting the message. Each key has a specific length of code. A strong encryption process requires a high key size [76]. In cryptography, private keys, session keys, and public kesy are frequently used. Below, different HAN models are discussed to illustrate how they used the cryptographic techniques.

In [55], a HAN sends its consumption to the NAN gateway, which is a trusted service provider and an interface between the HAN and utility provider. A NAN is distributed over a village, city, and sometimes over a residential or commercial area. The communication between the utility provider and the SM takes place via a gateway. The gateway should communicate with the SM in an offline mode. However, the proposed scheme establishes a session key using mutual authentication between the SM and gateway.

In HAN, appliances are arranged in two groups [77]. The first group is for one-way communication devices such as light bulbs, chargers, etc., while the second one consists of two-way communication appliances, e.g., electric vehicles, AC, etc. Before deployment, every smart appliance is assigned with an ID and master key. On the basis of the master key, the group header assigns a unique key and group controller key to every smart appliance and SM. The appliances encrypt their consumption using a unique key and send it to the group controller, which forwards it to the SM for further processing and verification. This scheme prevents man-in-the-middle attacks, Sybil attacks, and replay attacks, but ignores key updating.

Similarly, a cloud-based security scheme is proposed in [33] for smart homes, where home appliances are categorized into two different groups. Appliances which performs simple basic functions are placed in group 1. Group 2 contains controllable and monitoring devices which have two-way communication. Both groups have a group header. In this architecture, the SMs are not considered as the part of the smart home. The SM is considered as part of the AMI smart grid. Group headers are responsible for communicating with a home management system or cloud server. HMS is placed in a local cloud, which is controlled by a remote or simple device. Before deployment, every appliance and group header is assigned an ID. Using this ID, HMS generates a group key and shares it with the appliances and the group controller. Appliances use groups to further generate a unique key for communication inside the group. Every appliance before sending consumption or control signals, encrypts the data with unique key that is automatically generated by HMS.

IEC 61850 standard transmits a message in the time limit of 4 ms, which was more suitable than the existing schemes. To overcome the time bounded activity and privacy issues in existing schemes some proposals have been outlined. In [78], an authentication scheme is proposed, which comprises two phases: registration and key agreement. In the registration phase, a secure channel is established between the substation and data center, while in the key agreement phase—on the basis of a secure channel—unique session keys are created for communication and authentication. In the key agreement phase, the substation and data center authenticate each other and then a unique session key is established on the basis of passed parameters, i.e., certificate, ID, random number, and time stamp.

**Summary:**Table 4 presents the summary of key generation, key updating, and key sharing in [33,55,77,78,79] schemes. Schemes in [77,78] update their secret keys, but in the schemes discussed in [33,55,79], the secret keys remain the same.

### 4.5. Observations

This section highlights various shortcomings of the schemes discussed under data aggregation, CIA triad and anonymity, user authenticity, and key management. According to the findings, some issues are at hardware level and we need an international standard for the equipment used for the deployment of smart grids. Moreover, the hardware cost is another issue whereby the most important is that a majority of the proposed schemes discuss only one problem—e.g., authenticity or key management—and ignore other features, especially in the HAN context. If a proposed scheme does not consider the HAN model or some of the threads, then during the implementation phase of the smart grid, it can bring a number of severe issues such as cyber security concerns at the application level.

## 5. Proposed Scheme

In this section, we present a privacy preserving data aggregation scheme that employs the Chinese remainder theorem, one-way hash chain, and properties of modulo $n^{2}$ to aggregate the data [68,80,81].

### 5.1. System Model

In the proposed model, we consider smart home appliances, a dedicated sink node, a smart meter, and a third party trusted authority.

- Smart Home Appliances: Every smart appliance deployed in a HAN is equipped with sensing and communication equipment, which enables the sensing device to report its reading to the smart meter through a dedicated sink node. For simplicity, we can group home appliances based on their functionalities, e.g., lighting, fans, or kitchen appliances (microwave, refrigerator, toaster), etc. All devices with the same functionalities will be placed in the same group. There can be another grouping strategy such as by room with appliances as a separate group. Let us we have N number of appliances, AP. We can divide these appliances into *k* subsets such as $G_{1},G_{2},G_{3},G_{4}\ldots$$G_{k}$ where the size of subset $G_{i}$ is |$G_{i}$| = $G_{i}$. Since the smart appliances have limited computational power, we do not apply any time consuming and computationally extensive encryption algorithms.Therefore, lightweight security mechanisms are desired for smart appliances.
- Sink Node: First, we will make it clear, that we have a dedicated aggregator device with computational ability installed in each home. This dedicated device will be called a sink node. The sink node is really important as it acts as a relay device between home appliances and the smart meter. In particular, the sink node will aggregate the reading data from all the home appliances and forward the aggregated data to the smart meter. The sink node also applies some rules that help smart meters to identify any external attacks.
- Smart Meter: The smart meter receives the data from the sink node and does some data analytics. Since the data comes from heterogeneous devices, it is not appropriate to directly operate on all data. Therefore, the smart meter first calculates the mean and variance for each subset, which is a group of particular appliances. For the mean, we have the following equation:
  (1) $$M\left(G_{k}\right)=\sum_{G_{i}\epsilon G_{k}}x_{i}/N_{k};$$
  While for the variance, we can calculate it with:
  (2) $$Var\left(G_{k}\right)=\sum_{G_{i}\epsilon G_{k}}{x_{i}}^{2}/N_{k}-{M(G_{k})}^{2};$$
- Trusted Authority: Trusted authority is a trusted third party which initializes the system and manages key generation and other parameters for each entity in the network and assign keys to all the entities in the network including home appliances, sink nodes, and smart meters. Trusted authority will only be active while initiating the system and adding new appliances. It will will be offline afterwards. The trusted party will not participate in the following actions.

### 5.2. Threat Model

We assume that the trusted authority is a trusted third party and it will not be involved in any misconduct that can compromise the privacy of the HAN while the smart meter and sink node are honest but curious. The smart meter and sink node may be affected by undetected malware and those malware might eavesdrop on the smart appliances. The smart meter and sink node are honest, meaning that they will follow the design protocols. They are also curious, that is, they are also curious about smart appliance’s data privacy. They will not collude with each other. Smart appliances are not resourceful, so they are vulnerable to attacks. The attacks that might affect the smart appliances are false data injection by an external attacker or attacks may prevent a device from reporting readings or replay an old message. However, we have a resourceful smart meter and sink node, and the sink node will apply some techniques to check whether an appliance is malfunctioning or it is simply inactive at the time. If an appliance is inactive it will simply send a zero in its reading. The sink node can filter out the false data and will not include false data during the aggregation process.

### 5.3. Proposed Scheme

In this section, we present the proposed scheme, which consists of system initialization, appliance report, data aggregation, and analysis phases.

#### System Initialization

The trusted authority is a completely trustworthy party that starts the system. The trusted authority chooses two random prime numbers *m* and *n*, where *m = 2m’ + 1* and *n = 2n’ + 1* and |*m*| = |*n*| = $k_{0}$, compute *p = mn*, and λ = *lcm(m−1, n−1) = 2m’n’*; and defines a function, as in [82]

(3) L(x)=x−1/m

Then, consider that there are N home appliances inside a HAN. The trusted authority chooses *N + 2* random numbers such that

(4) $$\sum_{i=0}^{N+1}p_{i}=0mod\lambda$$

Suppose that there are k subgroups in the home area network and the maximum communications range for any group is [0, $X_{j}$], then we can define the range of data sensing for an appliance as *X = max($X_{1}$, $X_{2}$, $X_{3}$, …, $X_{k}$)*. Note that, the range [0, $X_{k}$] is a small message space as compared to $Z_{n}$. With this knowledge, the trusted authority chooses k + 1 prime numbers $\alpha_{0}$, $n_{1}$, $n_{2}$, $n_{3}$, *…*$n_{k}$, and computes

(5) $$\left\{\begin{array}{c} R=n_{1}\times n_{2}\times \cdots \times n_{k}\\ R_{i}=\frac{R}{n_{i}},\;y_{i}\equiv \frac{1}{R_{i}}\;\mathrm{mod}\;n_{i}\\ \sigma_{i}=R_{i}\cdot y_{i} \end{array}\right.$$

where all the prime numbers are of the same length, i.e., |$n_{i}$| = $k_{1}$ for 1≤i≤k. The condition of parameters is as follows (as taken from [82])

(6) $$\left\{\begin{array}{c} N\cdot X^{2}\leq \sigma_{0},\;N\cdot \left(X^{2}+X\cdot \sigma_{0}\right)<n_{i}\\ k_{1}\cdot \left(k+1\right)+\mathrm{lg}k<\left|n\right| \end{array}\right.$$

which enables us to gather all data in one cipher text. The trusted authority then chooses two secure hash functions *h, H* where h = (${0,1)}^{l}$ and H = (0,1)* ϵ$Z_{n}^{*}$ and a random number $t_{0}$ϵ (${0,1)}^{l}$ as the secret key. As the system initializes, the home appliance will report the power consumption periodically after a specific time. Thus, we divide the reporting time into w time slots for ease. At each time slot, the reporting appliance reports its reading and will send zero when a device is off or inactive. Thus, the trusted authority chooses a random number $t_{0}$ and generates a chain of N one way hash functions such as $HC_{1}$, $HC_{2}$, $HC_{3}$, *…*, $HC_{N}$ where each chain contains $HC_{N}$ = $h_{i1}$, $h_{i2}$, *…*, $h_{iw}$ which is of length (*w+1*), and $h_{iw}\epsilon ($0,1${)}^{l}$ is a randomly chosen number.

(7) $$h_{ij}=h\left(h_{i(j+1)}\|T_{j}\right)j=0,1,2,\ldots ,w-1$$

For each $h_{ij}$, 1 ≤ j ≤ w, the trusted authority will also compute its corresponding key

(8) $$key_{ij}=h\left(h_{ij}\|t_{0}\right)$$

The proposed scheme utilizes the property of a one-time password for authentication and encryption. For that purpose, we have $h_{ij}$ and $key_{ij}$ in time slot $T_{j}$. The header of each hash chain $h_{10}$, $h_{20}$, *…*, $h_{N0}$ will be signed with α by the third party trusted authority to ensure the validity of the hash chains for authentication. The scheme employs the AES algorithm for home appliances for encrypting the reading before sending it to the sink node. We have public parameters for the system, *parameter*: N, $n_{i}$: i = 1, 2, 3, …, k, $\sigma_{j}$: (j = 1, 2, 3, …, k), h, H, L(x), AES. Then, we have the public parameters for the system, so we will calculate key and assign it to the network entities.

- Every smart home appliance is assigned with a private key $p_{i}$, secret hash chain $HC_{i}$, corresponding keys *K* = $key_{i0}$, $key_{i1}$, $key_{i2}$, …, $key_{iw}$, and public *parameter*, which are shared over a secure channel.
- Then, a random number is chosen as the *shared key sk* between the sink node and the smart meter, which we assign to the sink node along with signed hash chain heads $h_{10}$, $h_{20}$, $h_{30}$, …, $h_{N0}$, α, and secret key $p_{N+1}$, $t_{0}$ and corresponding public *parameters* to the sink node.
- The smart meter is assigned the same key that is shared between the sink node and the smart meter and a secret key $p_{0}$, β, along with the public *parameters*.

### 5.4. Home Appliance Reporting

At every time slot $T_{s}$, each appliance will report its reading to the sink node by calculating the following:

- Step 1: Appliance uses its secret key $p_{i}$ and ($\sigma_{0}$, $\sigma_{j}$) to compute
  (9) $$c_{ip}\;=\;\left[1\;+\;n\cdot \sigma_{j}\cdot \left(x_{i}\cdot \sigma_{0}\;+\;x_{i}^{2}\right)\right]\cdot H{\left(T_{s}\right)}^{n\cdot p_{i}}\;\mathrm{mod}\;n^{2}\;$$
  and uses the key $key_{ip}$ to compute $C_{ip}$ = $AES_{key_{ip}}$($c_{ip}$). This method is used to prevent any external attacker from knowing the readings or to avoid the worst-case scenario that HAN might communicate with an unauthorized or compromised sink node.
- Step 2: $AP_{i}$ uses the hash value $h_{ip}$ from hash chain vector to compute
  (10) $$mac_{ip}=h(C_{ip}\left|\right|h_{ip})$$
- Step 3: The appliance then forwards the ($C_{ip}$, $h_{ip}$, $mac_{ip}$) to the sink node. The appliance can efficiently compute these parameters, especially if $H{\left(T_{s}\right)}^{n\cdot p_{i}}$ is computed in advance.

### 5.5. Sink Node Data Aggregation

Upon receiving the ($C_{ip}$, $h_{ip}$, $mac_{ip}$) in time slot $T_{s}$, the sink node checks whether the data is sent by an authenticated sender.

- Step 1: The sink node holds $h_{i0}$ from α; thus, the authenticity of each $h_{ij}$ on the hash chain $HC_{i}$ is simple to check. The sink node compares the freshness of the received hash value with the previously received hash values. If $h_{ip}$ has never been received before, $h_{ip}$ is accepted, otherwise, it is refused.
- Step 2: If $h_{ip}$ is valid, the sink node will compute $mac_{ip}^{\prime }$ by comparing it with the received value to check whether $C_{ip}$ has been altered or not.
  (11) $$mac_{ip}^{\prime }=h(C_{ip}\left|\right|h_{ip})$$
- Step 3: If $C_{ip}$ is accepted, the sink node computes $key_{ip}$ = h($h_{ip}$ || $t_{0}$) and uses $key_{ip}$ to reproduce $c_{ip}$ from $C_{ip}$ = $AES_{key_{ip}}$($c_{ip}$).

After verifying the encrypted readings received from all home appliances, the sink node runs the following data aggregation operation and calculates a single cipher text $C_{p}$ and sends ($C_{p}$, $mac_{p}$) to the smart meter.

(12) $$\left\{\begin{array}{c} C_{p}=\left(\prod_{i=1}^{N}c_{ip}\right)\cdot H{\left(T_{s}\right)}^{n\cdot p_{N+1}}\;\mathrm{mod}\;n^{2}\\ mac_{s}=h(C_{p}\left|\right|T_{s}\left||sk\right) \end{array}\right.$$

### 5.6. Smart Meter

Upon receiving the ($C_{p}$,$mac_{p}$) in time slot $T_{s}$, the smart meter verifies $mac_{p}$ using the shared key between the sink node and smart meter, as in [82]. Then, it validates the $C_{p}$. If $C_{p}$ is valid, the smart meter performs a report reading and analyses the received aggregated reading. Moreover, it calculates $H{\left(T_{s}\right)}^{n\cdot p_{0}}$ through its secret keys. Next, the smart meter computes using the following equations (as adapted from [82])

(13) $$C_{p}^{\prime }=C_{p}\cdot H{\left(T_{s}\right)}^{n\cdot p_{0}}\;\mathrm{mod}\;n^{2}$$

(14) $$C_{p}^{\prime }=\prod_{i=1}^{N}c_{ip}\cdot H{\left(T_{s}\right)}^{n\cdot (p_{0}+p_{N+1})}\;\mathrm{mod}\;n^{2}$$

(15) $$C_{p}^{\prime }=\prod_{i=1}^{N}\left[1\;+\;n\cdot \sigma_{j}\cdot \left(x_{i}\cdot \sigma_{0}\;+\;x_{i}^{2}\right)\right]\cdot H{\left(T_{s}\right)}^{n\cdot p_{i}}\;\mathrm{mod}\;n^{2}\;\cdot H{\left(T_{s}\right)}^{n\cdot (p_{0}+p_{N+1})}\;\mathrm{mod}\;n^{2}$$

(16) $$C_{p}^{\prime }=\prod_{i=1}^{N}\left[1\;+\;n\cdot \sigma {*}_{j}\cdot \left(x_{i}\cdot \sigma {*}_{0}\;+\;x_{i}^{2}\right)\right]\cdot \prod_{i=1}^{N+1}H{\left(T_{s}\right)}^{n\cdot p_{i}}\;\mathrm{mod}\;n^{2}$$

(17) $$C_{p}^{\prime }=\prod_{i=1}^{N}\left[1\;+\;n\cdot \sigma {*}_{j}\cdot \left(x_{i}\cdot \sigma_{0}\;+\;x_{i}^{2}\right)\right]\cdot H{\left(T_{s}\right)}^{n\cdot \sum_{i=1}^{N+1}p_{i}}\;\mathrm{mod}\;n^{2}$$

(18) $$C_{p}^{\prime }=\prod_{i=1}^{N}\left[1\;+\;n\cdot \sigma {*}_{j}\cdot \left(x_{i}\cdot \sigma_{0}\;+\;x_{i}^{2}\right)\right]\cdot H{\left(T_{s}\right)}^{n\cdot \lambda \cdot k}\;\mathrm{mod}\;n^{2}$$

(19) $$C_{p}^{\prime }=\prod_{i=1}^{N}\left[1\;+\;n\cdot \sigma {*}_{j}\cdot \left(x_{i}\cdot \sigma_{0}\;+\;x_{i}^{2}\right)\right]\;\mathrm{mod}\;n^{2}$$

(20) $$C_{p}^{\prime }=1\;+\;n\cdot \sum_{i=1}^{N}\left[\sigma {*}_{j}\cdot \left(x_{i}\cdot \sigma_{0}\;+\;x_{i}^{2}\right)\right]\;\mathrm{mod}\;n^{2}$$

(21) $$C_{p}^{\prime }=1\;+\;n\cdot \sum_{j=1}^{k}\sigma_{j}\left(\sum_{G_{i}\epsilon G_{j}}\left(x_{i}\cdot \sigma_{0}\;+\;x_{i}^{2}\right)\right)\;\mathrm{mod}\;n^{2}$$

The smart meter has the ability to calculate its mean and variance as

(22) $$\begin{array}{cc} M_{j}= & M\;\mathrm{mod}\;n_{j}=\sum_{i=1}^{N_{j}}\left(x_{i}\cdot \sigma_{0}+x_{i}^{2}\right) \end{array}$$

(23) $$\begin{array}{cc} E(G_{j})= & \frac{M_{j}-\left(M_{j}\;\mathrm{mod}\;\sigma_{0}\right)}{\sigma_{0}\cdot N_{j}} \end{array}$$

(24) $$\begin{array}{cc} Var(G_{j})= & \frac{M_{j}\;\mathrm{mod}\;\sigma_{0}}{N_{j}}-E{\left(G_{j}\right)}^{2} \end{array}$$

#### Fault Tolerance

In cases where an appliance is not reporting according to protocols, the sink node will aggregate the data received from other appliances and send the results to the smart meter and inform the smart meter that the appliance $AP_{a}$ is malfunctioning. The smart meter then uses the following method to calculate the mean and variance for the malfunctioning device.

- Step 1: $C_{p}^{*}$ is mathematically represented as
  (25) $$\begin{array}{cc} C_{p}^{*}= & \left(1+n\cdot \sum_{i=1,i\neq a}^{N}\sigma_{j}^{*}\cdot \left(x_{i}\cdot \sigma_{0}+x_{i}^{2}\right)\right)\\ & \cdot \prod_{i=1,i\neq a}^{N+1}H{\left(T_{s}\right)}^{n\cdot p_{i}}\mathrm{mod}n^{2} \end{array}$$
  Therefore, the smart meter computes
  (26) $$\begin{array}{cc} M_{s}^{*} & ={C_{p}^{*}}^{\lambda }\;\mathrm{mod}\;n^{2}\\ & \;\;\;\overset{{\left(1+n\cdot x\right)}^{\lambda }\equiv \left(1+n\cdot \lambda x\right)\;\mathrm{mod}\;n^{2},\;x^{\lambda n}\equiv 1\;\mathrm{mod}\;n^{2}}{\to }\\ & =1+n\cdot \lambda \cdot \sum_{i=1,i\neq a}^{N}\sigma_{j}^{*}\cdot \left(x_{i}\cdot \sigma_{0}+x_{i}^{2}\right)\;\mathrm{mod}\;n^{2} \end{array}$$
  and M can be calculated as
  (27) $$M=\left(\frac{M_{s}^{*}-1}{n\cdot \lambda }\;\mathrm{mod}\;n\right)\;\mathrm{mod}\;Q$$
- Step 2: Except for the subset containing malfunctioning devices, the smart meter calculates mean and variance using Equations (13) and (15).
- Step 3: For the subset containing malfunctioning devices, the smart meter computes $M_{b}$ from Equation (13) and gains the mean and variance through
  (28) $$\begin{array}{cc} E(G_{b})= & \frac{M_{b}-\left(M_{b}\;\mathrm{mod}\;\sigma_{0}\right)}{\sigma_{0}\cdot \left(N_{b}-1\right)} \end{array}$$
  (29) $$\begin{array}{cc} Var(G_{b})= & \frac{M_{b}\;\mathrm{mod}\;\sigma_{0}}{N_{b}-1}-E{\left(G_{b}\right)}^{2} \end{array}$$

Hence, the proposed approach is still workable even if some devices are malfunctioning. As a result, the proposed approach satisfies the need for fault tolerance.

## 6. Security Analysis

Here, we will determine that how the proposed approach can achieve the prevention of false data injection attacks and privacy preserving data aggregation.

### 6.1. Prevent False Data Injection

In the proposed scheme, the trusted authority uses one-way hash function, generates hash chains, and assigns a hash chain to each device for every time slot $T_{s}$. For every home appliance and for each time slot $T_{s-1}$, we have a hash value $h_{i(p-1)}$. From $h_{i(p-1)}$ = $h(h_{ip}||T_{s})$, we can authenticate $h_{ip}$ from $T_{s}$. However, because of the one-way nature of the hash function, we cannot obtain the $h_{ip}$ from $h_{i(p-1)}$. Moreover, since every device reports its reading directly to the sink node—and only if a device reports the correct data—we will receive the fresh $h_{ip}$ as we have assumed that the device will not act abnormally. If the $h_{ip}$ is not fresh in the time slot $T_{s}$, it means that it has been attacked and the false data is injected externally or the device is compromised and a replay attack has been launched externally. Therefore, the sink node will reject the false data. Thus, it is ensured that the proposed scheme is resistant to false data injection attacks.

### 6.2. Privacy Preserving

In the proposed scheme, if we consider the encrypted text by a home appliance

(30) $$\begin{array}{c} c_{ip}\;=\;\left[1\;+\;n\cdot \sigma_{j}\cdot \left(x_{i}\cdot \sigma_{0}\;+\;x_{i}^{2}\right)\right]\cdot H{\left(T_{s}\right)}^{n\cdot p_{i}}\;\mathrm{mod}\;n^{2}\; \end{array}$$

and that of the aggregated cipher text [82]

$$\begin{array}{cc} C_{p}^{*}= & \left(1+n\cdot \sum_{i=1,i\neq a}^{N}\sigma_{j}^{*}\cdot \left(x_{i}\cdot \sigma_{0}+x_{i}^{2}\right)\right)\cdot \prod_{i=1,i\neq a}^{N+1}H{\left(T_{s}\right)}^{n\cdot p_{i}}\;\mathrm{mod}\;n^{2} \end{array}$$

and take $\sigma_{j}\cdot \left(x_{i}\cdot \sigma_{0}\;+\;x_{i}^{2}\right)$ as a $\bar{message}$ and $\cdot H{\left(T_{s}\right)}^{\cdot p_{i}}\;$ as a random number $\bar{rand}$ and $\sum_{i=1,i\neq a}^{N}\sigma_{j}^{*}\cdot \left(x_{i}\cdot \sigma_{0}+x_{i}^{2}\right)$ as $\bar{Message}$ and $\prod_{i=1,i\neq a}^{N+1}H{\left(T_{s}\right)}^{\cdot p_{i}}$ as $\bar{Rand}$ then

(31) $$\begin{array}{c} c_{ip}\;=\;\left[1\;+\;n\cdot \bar{message}\right]\cdot {\bar{rand}}^{n}\;\mathrm{mod}\;n^{2}\; \end{array}$$

and

$$\begin{array}{c} C_{p}^{*}=\left(1+n\cdot \bar{Message}\right)\cdot {\bar{Rand}}^{n}\;\mathrm{mod}\;n^{2} \end{array}$$

are valid Paillier ciphers. Under the chosen plaintext attack, Paillier encryption is indistinguishable, and an external attacker cannot obtain the exact message. The sink node may be curious about the exact message. However, without the knowledge of secret keys $p_{0}$ and λ, the sink node has no knowledge of the information. Whereas the smart meter can recover $\bar{Message}$ and may want to recover the message sent by individual devices. For that purpose, it has to collude with the sink node, which is not possible under the threat model. Hence, the suggested technique protects aggregated data privacy.

## 7. Performance Evaluation

In this section, we analyze the communication and processing overhead of household appliances, sink nodes, and smart meters.

### 7.1. Communication Overhead

The proposed privacy preserving scheme aggregates the data from different subsets into one and the smart meter can recover the mean and variance of the individual subset. To demonstrate the efficiency of the proposed technique, we compare it to the basic Paillier encryption [80] in which the bit length of $n^{2}$ is 2048 and that of n is 1024. Therefore, the communication overhead from N devices to sink node is 2048 × N bits, because each home appliance encrypts both $x_{i}$ and $x_{i}^{2}$ into one cipher text, as shown in Equation (9). However, because the data in the BPE is encrypted into two cipher texts, the transmission cost is doubled, and the overhead is 4096 × N bits.

**Figure 2:**
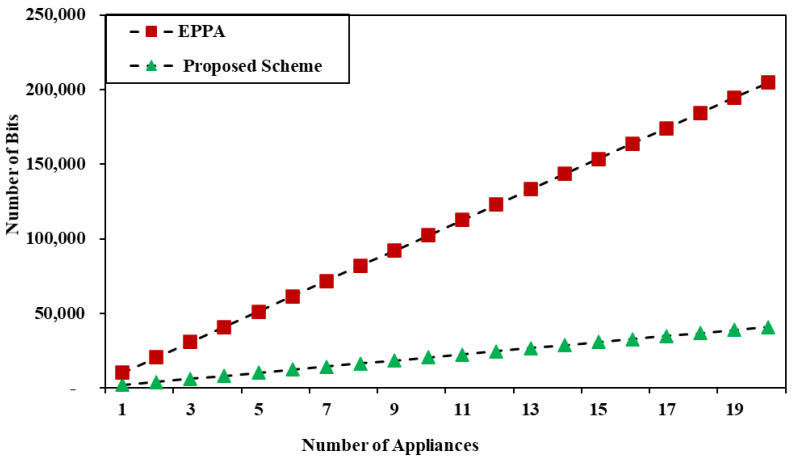
Communication overhead from home appliances to sink node.

**Figure 3:**
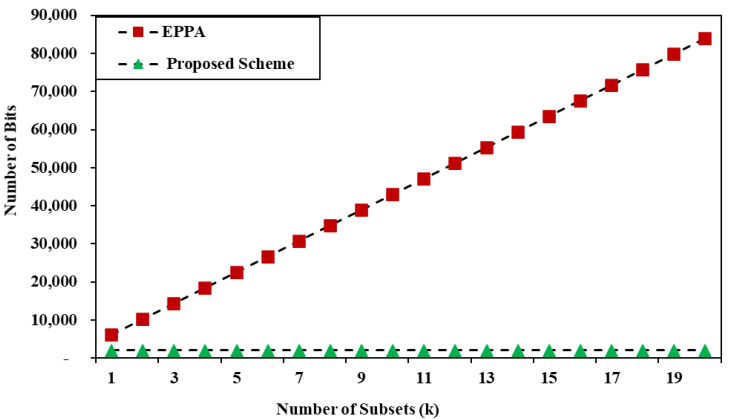
Communication overhead from sink node to smart meter.

In the proposed scheme, the sink node and smart meter are independent of the number of devices as the aggregation is undertaken at the sink node. In BPE, the communications cost is dependent on the number of subsets. If there are k subsets, then the communications cost is 4096 × k bits, while in the proposed scheme, all data have been aggregated in one cipher text. Therefore, the communications overhead from the sink node to the SM is only 2048 bits. Figure 2 plots a graph for the communications overhead from the home appliances to the sink node, and Figure 3 shows the communications overhead pattern of the sink node to the SM. As a result, it is obvious that the suggested method is superior to BPE in terms of communication costs. We use the Chinese remainder theorem that enables a careful parameter choice and, hence, in real-time scenarios the message size is small.

### 7.2. Computation Overhead

The suggested system is lightweight in terms of communication costs since we only use the time-consuming modulo operation, and each node in the network, such as a sink node, a smart meter, and a home appliance, has at least one modulo operation. The proposed approach will become more efficient if the modulo exponent is computed ahead of time.

## 8. Future Directions and Challenges

In this paper, we have discussed smart grids and the relation among different entities of the smart grid and how they interact with each other. After that, we reviewed the literature regarding countermeasures to the posed threats and discussed some promising solutions that are suggested in order to overcome privacy related issues and threats. Lastly, we proposed a novel privacy preservation scheme for HANs. To complete the effort, we have devised a direction for future research and challenges, which are discussed below.

### 8.1. A Safe and Secure Trust Mechanism for Home Incumbents in Smart Grid

As discussed in Section 1, smart grids work through the coordination of different entities. The smart gird is a network of different entities and different subnetworks working together. Each entity and subnetwork has its own requirements. The continuous communication is essential to ensure the smart grid remains active. Iinteroperable and uninterrupted communication between the different subnetworks is an intimidating task. Therefore, a universal standardized trusted framework is essential for any communication.

### 8.2. Government Authorities to Regulate and Maintain Smart Grids

Most of the research work performed is voluntary and in order to make smart grids a success, it is necessary to have a government authority to evaluate the standard and conformity of the research undertaken on smart grids. Thus, the authorities can make the necessary decisions and improvements needed to regulate smart grids.

### 8.3. New Goals and Standards to Evaluate Privacy Preserving Mechanism and Solution

The authorities should set standards and new metrics to evaluate any new research or protocols. Each new research idea should be evaluated on common standards and then a decision should be made whether to make it a standard or revise an old standard.

### 8.4. Legal Code for Preserving the Privacy

A legal framework through the contributions of both governments and business authorities should be made to protect the privacy of consumers and other network entities. The legal framework would help to set the standard to what extent a user’s data can be collected and how it can be manipulated to further increase the efficiency of the smart grid. In cases of breach of this contract, the legal framework should outline what the consequences would be faced by the entity breaching the contract.

### 8.5. A Framework for Aggregation without Third Party Involvement

The third-party involvement in the aggregation can be compromised anytime. A scheme should be devised that could aggregate the data without knowing the meaning of the aggregated data that could not harm or lead the smart grid to instability.

## 9. Conclusions

We proposed a novel privacy-preserving data aggregation approach for HANs in a smart grid in this paper. The proposed approach deploys a sink node between the household appliances and the smart meter, which not only filters false data injection attacks but also provides for early fault tolerance. The technology also combines data from several home appliances, which may belong to distinct subsets, into a single stream and sends it to the smart meter. The suggested technique is secure, and the performance evaluation reveals that it is more efficient in terms of communications and computation overhead than aggregation using basic Paillier encryption. Detecting and avoiding new threats, IDS architectures for smart grid privacy, IoT-driven smart grids, new privacy metrics, and privacy for IoT are all demanding research fields that need to be further explored in the future.

## Figures and Tables

**Table 1:** Comparison of data aggregation schemes.

Schemes	Technique Used	Appliances Aggr:	SM Aggr:	Separate Device for Aggr:	CC Aggr:	Descriptions
[67]	MPC	No	No	No	Yes	**Pros:** Used multiparty computation scheme, universal composition, deals multiple recipients, create subsets of SMs, and fault tolerant. **Cons:** Cost of communication server and communication overhead.
[22]	PPCR	No	No	Yes	Yes	**Pros:** Incremental-hash function used and only cost of consumption is circulated. **Cons:** Aggregation performed by an outside device, i.e., operation center. Did not address various attacks, key generation, and authenticity.
[65]	Distributed aggregation	No	No	Yes	No	**Pros:** Grouping, each group has an aggregator, and slices send randomly to the multiple group aggregator. **Cons:** Creation of groups, selection of group header, reassembling of various slices at the CC end, and communication overhead.
[30]	In-network aggregation	Yes	No	No	No	**Pros:** Before installation devices are authenticated, hop-by-hop aggregation. **Cons:** Creation of chip codes, computation burden, and no procedure to update key.
[66]	MLTD	No	No	Yes	Yes	**Pros:** Blinding factor generated by TTP, provides unforgeability, resistant to MIMT, alteration, and spoofing. **Cons:** A separate device for aggregation is an issue, provides computation and communication overhead, and did not provide key generation and updating process.

**Table 2:** Comparative analysis of CIA models and anonymity.

Ref.	Method	Confidentiality	Integrity	Availability	Anonymity	Description
[68]	PPMA	Yes	Yes	No	No	**Pros:** Uses Paillier cryptography, provides individual privacy, and creates parameters for key generation. **Cons:** Shares key parameters on wireless media, uses external device for aggregation, and does not discuss availability and various attacks.
[69]	LiPSG	Yes	Yes	No	No	**Pros:** Uses Q-learning, good at computation, and splits energy consumption into two subsets. **Cons:** Depends on third party server, does not discuss attacks, and has no aggregation point.
[16]	Lattic based scheme	Yes	Yes	No	No	**Pros:** Aggregation performed by every device in rounds, every sender is authenticated by receiver, and except CC, no one can decrypt consumption. **Cons:** Outside network third party issues appliance ID and generates keys and the key remains the same.
[21]	Blind signature	Yes	Yes	Yes	No	**Pros:** Creation of blocks, shares key material instead of secret keys, and provides anonymity and traceability. **Cons:** Ignores smart appliances, has no mechanism for key updating, has no verification of authenticity inside homes, and computes overhead at CC end.
[70]	LWPPS	Yes	Yes	No	No	**Pros:** Shares key material instead of private key, does not involve a third party, and performs encryption and decryption at the CC end. **Cons:** CC sends request message, communications overhead, and does not explain threat model and authenticity.

**Table 3:** Summary of authentication schemes.

Scheme	Appliances Auth:	SM Auth:	CC Auth:	Descriptions
Schnorr’s signature [72]	No	No	Yes	**Pros:** Uses Schnorr’s signature for authentication and key agreement, low communications and computation cost, and updating secret as well as session keys after intervals. **Cons:** The process of registration is performed by third party, TA. It does not discuss HAN and appliances. No mechanism is defined for aggregation.
EFFECT [15]	No	Yes	Yes	**Pros:** Introduces a threshold value for data aggregation. Provides integrity, authenticity, and availability. **Cons:** The TCA sets up the whole architecture, is responsible for key generation, aggregation, and selection of gateways. Increases computation by using secret sharing.
PUC [73]	No	Yes	No	**Pros:** Prevents inside and outside attacks. Detects any attack easily using PUF. **Cons:** The whole functioning of SM fails if anyone tries a physical attack. Has an expensive chip and lengthy process of initial registration.
Blind signature [21]	No	Yes	Yes	**Pros:** Creates blocks, shares key material instead of secret keys, and provides anonymity and traceability. **Cons:** Ignores smart appliances, no mechanism for key updating, authenticity is verified outside the home, and computation overhead at the CC end.
ECCAuth [74]	No	Yes	Yes	**Pros:** Proposes real model with mutual authentication of devices locally and globally. **Cons:** High in computation and does not address anonymity and denial of service attacks.
IBS [75]	No	Yes	Yes	**Pros:** Minimum role of TTP. TTP is only involved in initial registration, key generation, and parameter creation. Provides anonymity, authenticity, traceability, and confidentiality. **Cons:** Does not explain how to update the private key, aggregation, and DDos attacks. Requires higher computation resources and scalability.

**Table 4:** Comparative analysis of key management schemes.

Scheme	Key Generation	Key Sharing	Key Updating	Description
COT [77]	Yes	No	Yes	**Pros:** IoMT-based HAN structure, based on mesh topology with a single source of energy collection. **Cons:** Selection of header for group and TA authenticate home appliances.
LAKA [55]	Yes	Yes	No	**Pros:** Lower communication overhead, provides mutual authentication, and key generation and anonymity. **Cons:** Ignores appliances operations and no key updating and traceability.
KMP [79]	Yes	No	No	**Pros:** Uses elliptic curve, provides session key agreement, and used less communication and computation overhead. **Cons:** Involves a third party for authentication, no key updating, traceability, and anonymity.
IEC [78]	Yes	No	Yes	**Pros:** Updates IEC 62,351 standard with respect to symmetric key encryption and creates session key with a transmission of message within 4 ms. **Cons:** No mechanism for data aggregation or communication overhead.
COT-HAN [33]	Yes	Yes	No	**Pros:** The HAN architecture is based on HMS and cloud based infrastructure and a cell phone has the authority to access cloud services. **Cons:** SM is not considered part of the HMS; no proper structure for authentication and the symmetric key value remains the same; vulnerable to MIMA, DDoS, and alteration attacks; and no aggregation point.

## Abbreviations

**Abbreviation**	**Definition**
ADDM	Distributed State Estimation
Aggr:	Aggregator
Auth:	Authentication
BAN	Building area network
BPE	Basic Paillier Encryption
CC	Control center
CIA	Confidentiality, integrity, and availability
COT	Clouds-of-things
COT-HAN	COT home-area-network
DDOS	Denial-of-service attack
DR	Demand respond
EPPA	Efficient Privacy-Preserving Aggregation
HAN	Home-area-network
H-MAC	Hashed based MAC
HMS	Home management system
IBS	Identity based signature
ID	Identification
KMP	Key-Management protocol
LAKA	Lightweight Authentication key agreement
LPPS	Lightweight privacy preservation
LWPPS	Lightweight-privacy-preserving
MAC	Message authentication code
MLAN	Metropolitan LAN
MLTD	Multidimensional aggregation
MPC	Multiparty computations
NAN	Neighbour area network
PALK	Password-based anonymous lightweight key
PK	Private key
PPCR	Privacy-preserving cheat resilient
PPMA	Privacy-preserving multi-subset
PS	Power supplier
PUC	Physical unclonable function
SA	Smart Appliance
SG	Smart grid
SM	Smart meter
SP	Service provider
TAI	Threshold-Based Anonymous Identification
TTP	Trusted third party
WAN	Wide area network
